# Supplementary figures and images for: Population Coupling of V1 and V4 Neurons and Its Relation to Local Cortical State Fluctuations and Attention in Macaque Monkey
Source: eNeuro. 2026 May 8;13(5):ENEURO.0091-26.2026. doi: 10.1523/ENEURO.0091-26.2026 (PMC13171287; doi:10.1523/ENEURO.0091-26.2026)

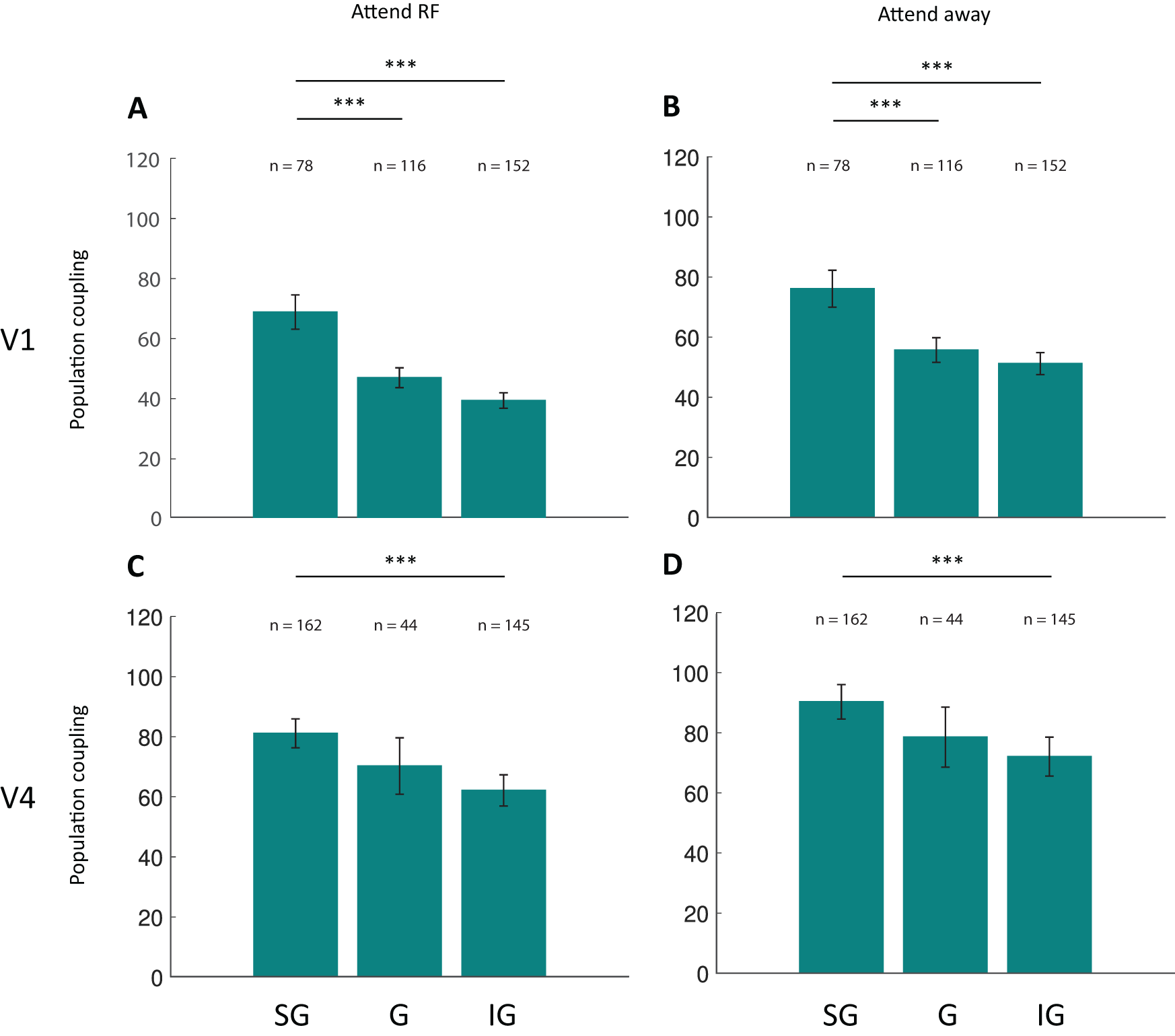

Supplement: Figure 2-1 — Population coupling in supragranular (SG), granular (G) and infragranular (IG) layers. A) Population coupling during attend RF stimulus driven activity for neurons located in different layers of V1. B) Population coupling during attend away stimulus driven activity for neurons located in different layers of V1. C) Same as A, but for V4. D) Same as C but for V4. Statistics: Kruskal-Wallis test (Bonferroni corrected); data are represented as means ± SEMs; significance levels *p < 0.05, **p < 0.01, and ***p < 0.001. Download Figure 2-1, TIF file. [file eneuro-13-ENEURO.0091-26.2026-s001.tif]

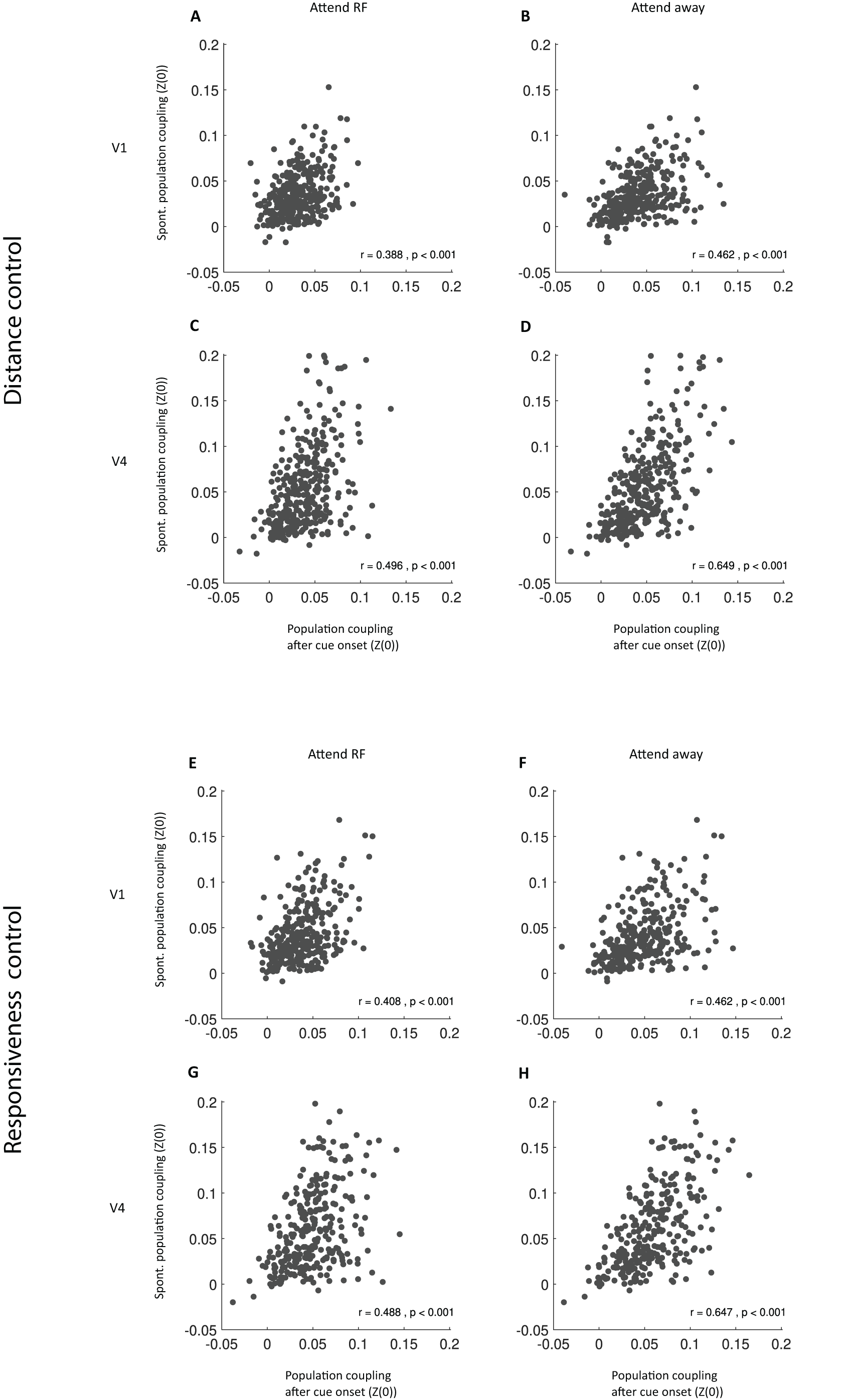

Supplement: Figure 3-1 — Population coupling during spontaneous activity compared to stimulus driven activity (PC quantified by calculating the Fisher transformed correlation coefficient Z(0) between single unit and population activity). A) Population coupling during spontaneous vs. attend RF stimulus driven activity for V1 neurons (period after cue onset). B) Population coupling during spontaneous activity vs. attend away stimulus driven activity for V1 neurons (period after cue onset). C) same as A) but for V4 neurons. D) Same as B) but for V4 neurons. E-H shows the same comparisons as A-D, but for responsiveness controls. A-D shows ‘distance/leakage’ controls (excluding channels less than 200um apart in population activity calculation). E-H shows responsiveness controls (including only single units that responded significantly to stimulus presentations. Insets show correlation coefficient between the two measures, and respective p-values. Download Figure 3-1, TIF file. [file eneuro-13-ENEURO.0091-26.2026-s002.tif]

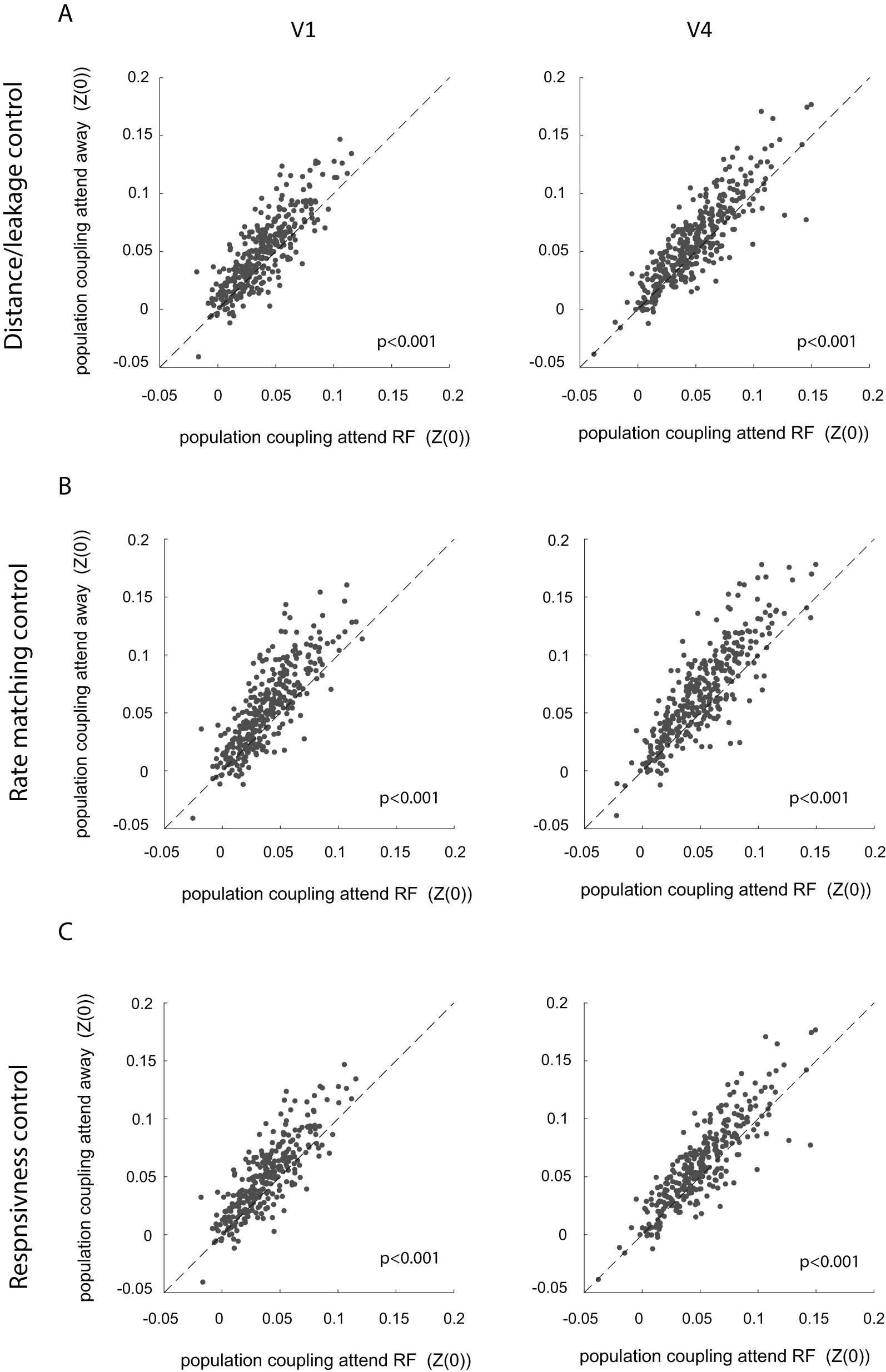

Supplement: Figure 4-1 — Effect of attention on population coupling of V1 and V4 neurons. Population coupling (Fisher-transformed correlation coefficient (Z(0)) during attend RF conditions (x-axis) and attend away conditions (y-axis) during the period after cue-onset for V1 neurons (left) and V4 neurons (right). A) shows controls for leakage by excluding channels less than 200um apart in population activity calculation. B) shows rate matching controls. C) shows controls for responsiveness. Insets show p-values of differences in population coupling. Download Figure 4-1, TIF file. [file eneuro-13-ENEURO.0091-26.2026-s003.tif]

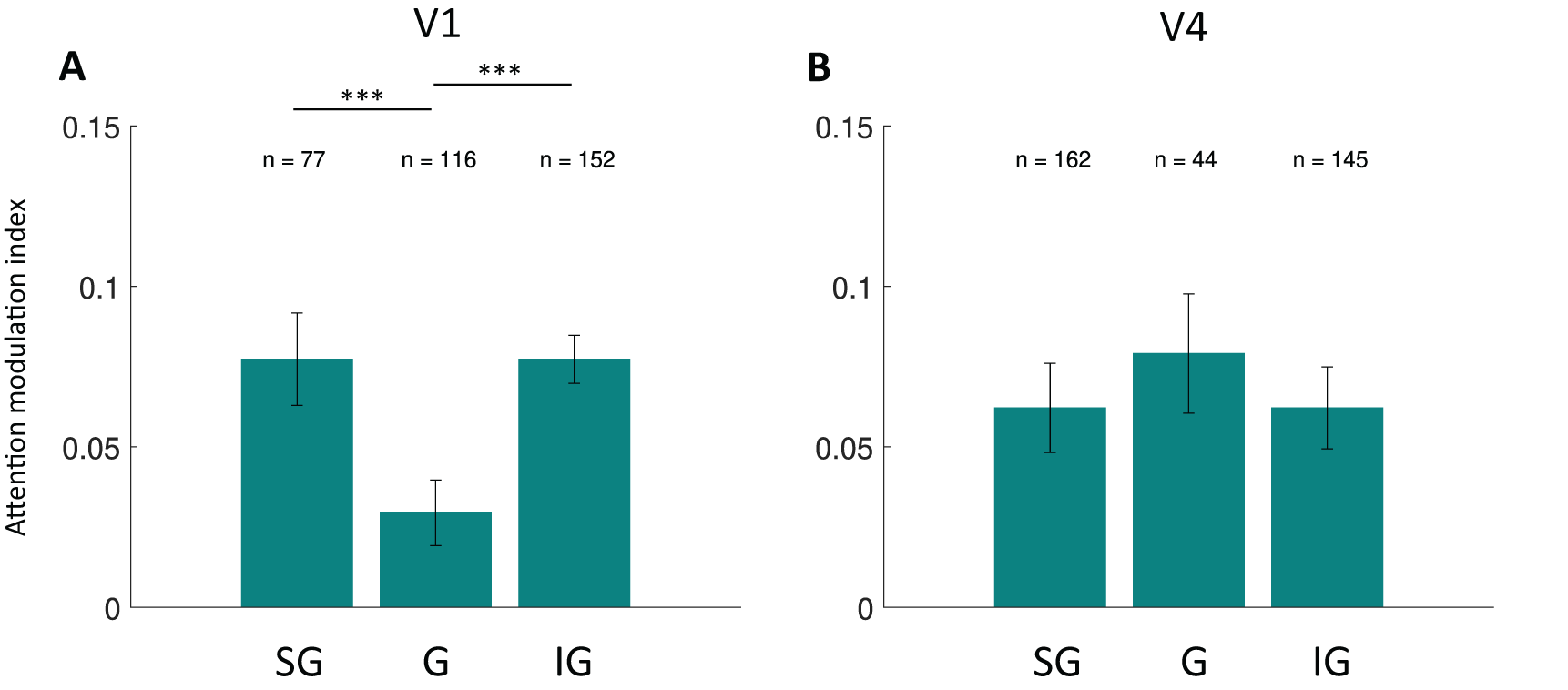

Supplement: Figure 4-2 — Attentional modulation indices in supragranular (SG), granular (G) and infragranular (IG) layers. A) Attention modulation for neurons located in different layers of V1. B) Attention modulation for neurons located in different layers of V4. Statistical test: Kruskal-Wallis test (Bonferroni corrected); data are represented as means ± SEMs; significance levels *p < 0.05, **p < 0.01, and ***p < 0.001. Download Figure 4-2, TIF file. [file eneuro-13-ENEURO.0091-26.2026-s004.tif]

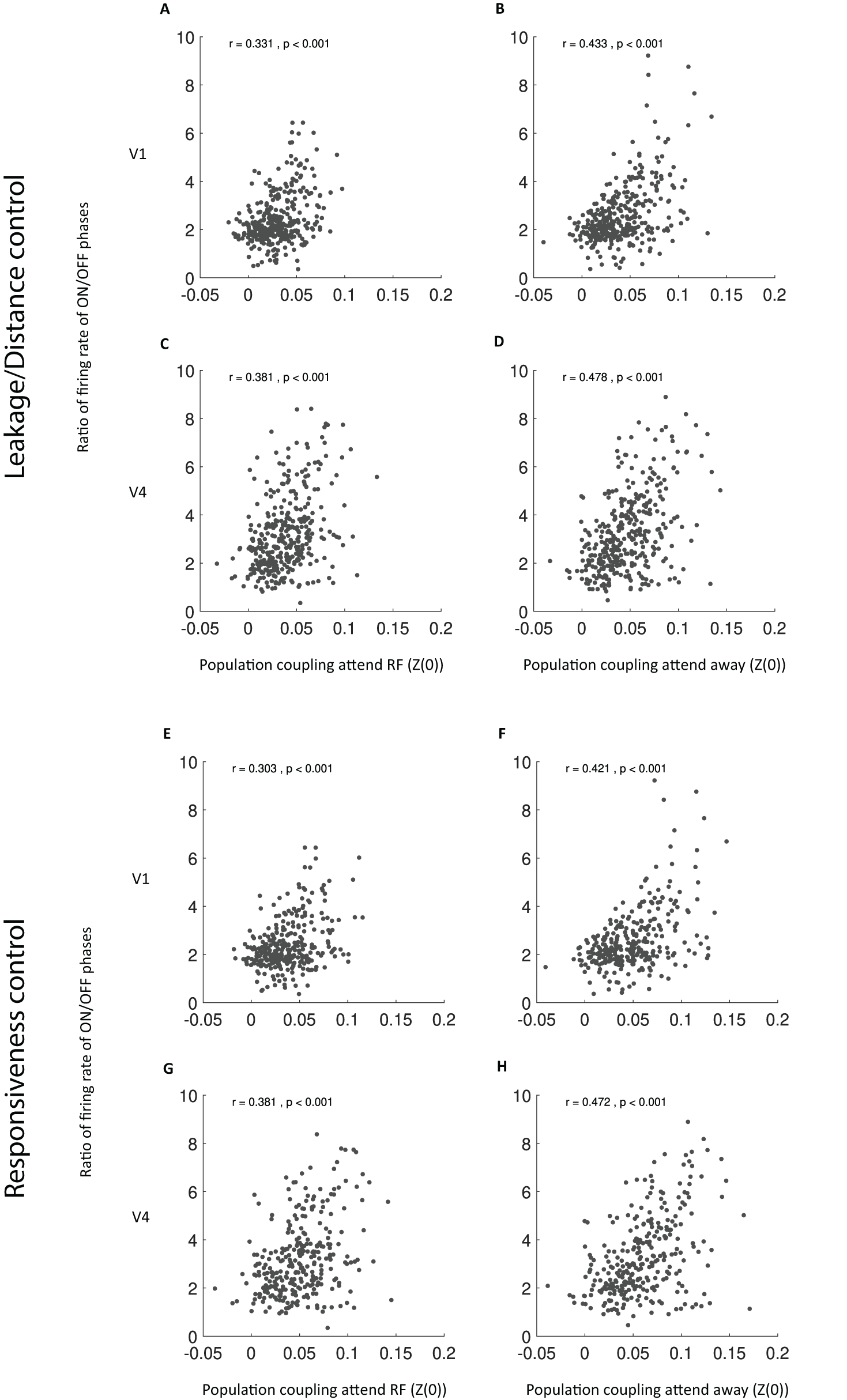

Supplement: Figure 5-1 — ON-OFF state fluctuation alignment and its relation to population coupling of V1 and V4 neurons. Population coupling (Fisher-transformed correlation coefficient (Z(0)) during attend RF conditions and attend away conditions during the period after cue-onset for V1 neurons (left) and V4 neurons (right) compared to the ratio of ON-OFF period firing (y-axis). A-D shows leakage/distance controls, excluding channels less than 200um apart in population activity calculation. E-H shows responsiveness controls, including only neurons that responded significantly to stimulus presentations, Statistic: Wilcoxon signed rank test. Insets show correlation coefficients and p-values. Download Figure 5-1, TIF file. [file eneuro-13-ENEURO.0091-26.2026-s005.tif]

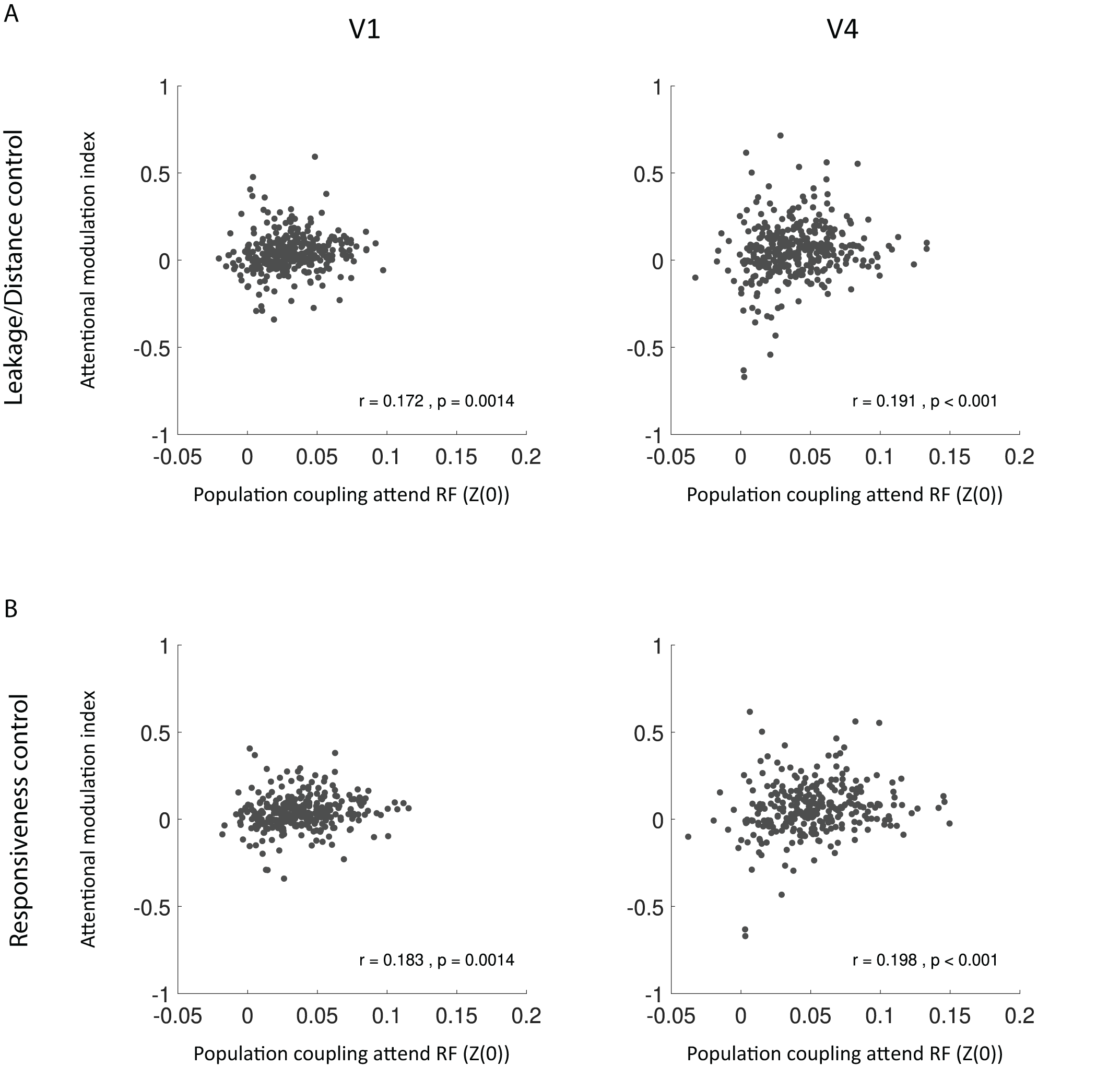

Supplement: Figure 6-1 — Relation between population coupling and attentional modulation of V1 and V4 neurons. Population coupling (Fisher-transformed correlation coefficient (Z(0)) during attend RF conditions during the period after cue-onset for V1 neurons (left) and V4 neurons (right) compared to the strength of attentional modulation (y-axis). A) shows leakage/distance controls, excluding channels less than 200um apart in population activity calculation. B) shows responsiveness controls including only neurons that responded significantly to stimulus presentations, Statistic: Wilcoxon signed rank test. Insets show correlation coefficients and p-values. Download Figure 6-1, TIF file. [file eneuro-13-ENEURO.0091-26.2026-s006.tif]
